# P16^INK4a^ Regulates ROS-Related Autophagy and CDK4/6-Mediated Proliferation: A New Target of Myocardial Regeneration Therapy

**DOI:** 10.1155/2023/1696190

**Published:** 2023-02-18

**Authors:** Jiateng Sun, Liuhua Zhou, Tongtong Yang, Bo Deng, Yulin Bao, Lingfeng Gu, Hao Wang, Liansheng Wang

**Affiliations:** Department of Cardiology, The First Affiliated Hospital of Nanjing Medical University, Nanjing 210029, China

## Abstract

Neonatal mice achieve complete cardiac repair through endogenous myocardial regeneration after apical resection (AR), but this capacity is rapidly lost 7 days after birth. As an upstream inhibitor of cyclin-dependent kinase 4/6- (CDK4/6-) mediated cell cycle activity, p16^INK4a^ is widely involved in regulating tumor and senescence. Given that p16^INK4a^ had a significant negative regulation on cell proliferation, targeting cardiomyocytes (CMs) to inhibit p16^INK4a^ seems to be a promising attempt at myocardial regeneration therapy. The p16^INK4a^ expression was upregulated during perimyocardial regeneration time. Knockdown of p16^INK4a^ stimulated CM proliferation, while p16^INK4a^ overexpression had the opposite effect. In addition, p16^INK4a^ knockdown prolonged the proliferation time window of newborn myocardium. And p16^INK4a^ overexpression inhibited cell cycle activity and deteriorated myocardial regeneration after AR. The quantitative proteomic analysis showed that p16^INK4a^ knockdown mediated the cell cycle progression and intervened in energy metabolism homeostasis. Mechanistically, overexpression of p16^INK4a^ causes abnormal accumulation of reactive oxygen species (ROS) to induce autophagy, while scavenging ROS with N-acetylcysteine can alleviate autophagy and regulate p16^INK4a^, CDK4/6, and CyclinD1 in a covering manner. And the effect of inhibiting the proliferation of p16^INK4a^-activated CMs was significantly blocked by the CDK4/6 inhibitor Palbociclib. In summary, p16^INK4a^ regulated CM proliferation progression through CDK4/6 and ROS-related autophagy to jointly affect myocardial regeneration repair. Our study revealed that p16^INK4a^ might be a potential therapeutic target for myocardial regeneration after injury.

## 1. Introduction

Up to now, ischemic heart disease has been the leading cause of disability and mortality worldwide [1]. Since cardiomyocytes (CMs) are nonrenewable, the focus of previous research is to save the dying CMs after myocardial injury. In recent years, with the discovery of the mammalian myocardial regeneration time window, it has become possible to do incremental research on endogenous myocardial regeneration in addition to the research on the CMs preservation after injury due to various physicochemical factors [2]. Neonatal mice can achieve complete morphological and functional recovery after apical resection (AR) within 7 days postborn, but this regeneration capacity is rapidly lost afterwards [3]. It is generally believed that the loss of proliferative capacity and the terminal differentiation state represents the decisive endpoint of the developmental program. During the neonatal period of mammalian CMs, reactive oxygen species (ROS) accumulation in CMs is caused by environmental oxygen concentration, which leads to DNA oxidative stress damage, ultimately resulting in persistent cell cycle arrest and failure to respond to mitotic stimuli [4].

For eukaryotic cells, cell cycle regulation in proliferation mainly occurs at two key points: G1-S and G2-M. The G1-S phase is the highest trigger point in cell cycle progression [5]. In the G1 phase, cells prepare for genome replication and receive signals promoting self-renewal, differentiation, or cell cycle exit [6]. The G1 to S transition is regulated by various factors, among which cyclin-dependent kinases (CDKs) regulate transcription and replication to preserve genome integrity and coordinate changes in cell adaptation and development. Therefore, it is regarded as a direct regulatory participant by many researchers and has been deeply explored [7]. DiStefano et al. found that transfection with endogenous CDKs inhibitors (e.g., p21 (Waf1), p27(Kip1), and p57(Kip2)) can reactivate neonatal and adult CMs into the cell cycle. The combined expression of CDK1, CDK4, CyclinB1, and CyclinD1 induces myocardial nuclear replication and division in mice, rats, and humans and achieves myocardial proliferation through stable cytokinesis [8]. Therefore, it is an exciting research direction to reenter the CM proliferation cycle by intervening CDKs family.

In mammals, CDKi is divided into two categories. The INK4 family specifically binds and inhibits CDK4/6, including p16^INK4a^, p15^INK4b^, p18^INK4c^, and p19^INK4d^. CIP/KIP family includes p21^CIP1^, p27^KIP1^, and p57^KIP2^ [9]. Although CIP/KIP protein is generally used as a negative regulator of Cyclin E, Cyclin A-CDK2, and Cyclin B-CDK1 holoenzyme, they also act as a positive regulator of Cyclin D-CDK4/6 complex by mediating its assembly at the early stage of G1 [10]. In previous studies, p21^CIP1^ and p27^KIP1^ in regulating CM proliferation have been clarified, but the role of the INK4 family has not been entirely concerned [11]. The four proteins of the INK4 family have similar structures and are dominated by several anchor protein repeats. Although the INK4 family members seem redundant in structure and equally effective as inhibitors, the INK4 family members express differently during mouse development. The significant diversity of INK4 gene expression patterns indicates that the cell cycle inhibitor family may have cell-specific or tissue-specific functions [12]. It is still unknown that p16^INK4a^ plays a specific role in myocardial regeneration and repair involving cardiomyocytes. Somatic cell-specific loss of p16^INK4a^ through point mutation or small deletion has been reported in thousands of human cancers. Similarly, 56 different germline mutations that only target p16^INK4a^, and retain p14^ARF^ and p15^INK4b^, have been described in unrelated kindreds that are cancer-prone [13]. Coincidentally, unlike other members of the INK family, p16^INK4a^ expression can be used as a biomarker of physiological age. Its overexpression reduces the replicative potential of specific self-renewing departments with ageing [11]. In addition, p16^INK4a^ has homology in adult CMs with proliferative ability [14]. Therefore, the study of INK4a is much more promising than ARF and INK4b in investigating the reactivation of the proliferation cycle of mammalian CMs.

In previous studies, cooverexpression of p16^INK4a^ and p21^WAF1^ inhibited Ang II-induced CMs hypertrophy [15]; p16^INK4a^ knockdown inhibited the PDGFB and TWIST1 expression in pulmonary arterial hypertension and subsequent vascular remodelling [16]. In addition, our previous research demonstrated that timely p16^INK4a^ overexpression could restrain the proliferation of cardiac fibroblasts, thus inhibiting the myocardial fibrosis process [17]. However, whether p16^INK4a^ functions in CMs during the regenerative repair deserves further investigation. At the same time, p16^INK4a^ is involved in cell proliferation and senescence through the cell cycle regulation, so it is regarded as a potential therapeutic target for the exploration of new targeted drugs and as a marker of cell senescence in the field of oncology [18, 19]. Cell senescence is a specific phenomenon in which proliferative cells experience permanent growth arrest in response to various cellular stresses [20]. As the beginning and end of CM fate, the proliferation withdrawal and senescence initiation seem to reach tacit agreement in the cell cycle regulation. Therefore, p16^INK4a^, widely recognised in cell senescence, has a unique and intriguing significance in regulating the time window of myocardial regeneration.

## 2. Materials and Methods

### 2.1. Animal

The Institute of Cancer Research (ICR) pregnant mice and neonatal 1-day-old mice used in this study were purchased and raised in the Animal Center of Nanjing Medical University. The mice used in the experiment were kept in standard pathogen-free SPF animal facilities in the Laboratory Animal Center of Nanjing Medical University, with light and dark cycles of 12 : 12, constant temperature of 20-22°C, constant humidity of 50%-70%, and adequate water and food. All animal experiments involved in this study were approved by the Animal Management and Use Committee and authorised by the Animal Ethics Committee of Nanjing Medical University (No. 1601038). The animals involved in anaesthesia, surgery, and other operations follow the relevant regulations of Jiangsu Province Experimental Animal Management Measures (Jiangsu Provincial People's Government No. 45).

### 2.2. Apical Resection and Virus Injection into the Myocardium at the Edge of the Excision Area

Neonatal mice (1-day postnatal, P1) were collected and placed in a separate cage. P1 mice were anaesthetised at a low temperature (hypothermia can provide anaesthesia by reducing nerve conduction and synaptic transmission). Subsequently, the mice were placed under a stereomicroscope, and the skin was disinfected with a cotton swab dipped in betadine. Microsurgical scissors cut the skin from left to right between the third and fourth ribs of mice, and the rib cage was exposed after the blunt separation of the intercostal muscles. Surgical scissors removed about 15% of the apex tissue from the left ventricle. After surgery, the heart was gently returned to the chest using a saline swab, and the ribs and skin were closed layer by layer using surgical sutures (6-0). Finally, the mice were placed under an infrared physiotherapy lamp to restore body temperature. When the skin was rosy and the limbs began to move, the mice were put back in the cage. After the operation of all mice, all mice were sent to the mother mice. The sham group performed the same procedure described above without AR. A specific scheme of combined adenovirus injection in AR of mice (AR + Ad5-cTNT-INK4ai or AR + Ad5-cTNT-NC): 10% Trypan blue solution with PBS, and adenovirus stock solution was diluted 1 : 9 in Trypan blue solution (the total amount of injected virus was 1^∗^10^9^ pfu/virus). A microsyringe with a 36 G needle was used to drain the virus diluent. The needle was injected slowly at three locations around the apex of the heart (anterior, lateral, and posterior wall: 2 *μ*l each). Under the microscope, the edge of the myocardium can be seen to be stained blue, indicating successful injection.

### 2.3. Echocardiography

Cardiac function was assessed by echocardiography 1 day before surgery and 28 days postresection (28 dpr) (AR + Ad5:cTNT-INK4ai vs. AR + Ad5:cTNT-CON). All echocardiographic tests were performed at the Animal Laboratory Center of Nanjing Medical University in this study. The mice were first anaesthetised by inhalation, and then, the left ventricular ejection fraction (EF) and left ventricular fractional shortening (FS) of each mouse were measured by small animal echocardiography. Finally, the left ventricular systolic function parameters (EF/FS) were calculated by accompanying software.

### 2.4. Masson Staining and Assessment of Infarct Size

For Masson staining, we collected the 28 dpr hearts of mice. Then, it was fixed overnight in 4% paraformaldehyde and soaked in paraffin for embedding. The paraffin blocks were fixed and sliced with a thickness of 5 *μ*m. Following standard procedures, the sections were dewaxed and rehydrated with gradient ethanol via the Masson staining kit (Solarbio G1340, Beijing). Under the microscope, the normal myocardium is red, and the scar area is blue. Finally, the Image J software was used to calculate the scar area and left ventricular zone, and the percentage of scar area/left ventricular zone was statistically analysed.

### 2.5. Neonatal Mice Cardiomyocytes (NMCMs) Isolation, Culture, and Transfection

NMCMs were extracted from 50 1-day-old neonatal mice each time (a total of about 200 newborn mice). After being disinfected with 70% ethanol for 3 s, the heart was immediately removed and placed in precooled 1 × ADS. Residual blood was squeezed from the heart cavity, and the isolated heart was transferred to the new 1 × ADS. After all the hearts were collected, the sterile flask was transferred to the superclean table. The scissor was used to cut the tissue thoroughly. The myocardial tissue fragments were resuspended with an appropriate amount of preheated myocardial tissue digestive fluid, then transferred to a 100 ml sterile bottle with a fixed volume of 20 ml digestive fluid, and digested in a constant temperature level shaker for 8-10 minutes. The supernatant containing CMs was taken, and 4 ml HS was added to terminate digestion and buried in ice for resting. Another 20 ml of myocardial digestive fluid was added, and the above steps were repeated until all myocardial tissue was wholly digested. The digested liquid was centrifuged, the supernatant was discarded, and the CM medium was added to resuspend the precipitation. Then, filter the suspensions and remove fibroblasts and incomplete digested myocardial tissue. Subsequently, the obtained CM suspension was evenly distributed in the dish and placed in a constant temperature incubator with differential adhesion for 90 minutes. The culture medium was collected and carefully cleaned. After centrifugation, an appropriate amount of 1 × ADS was added for resuspension. Density gradient centrifugation was performed using percoll solution. CMs distributed in the middle layer were collected and cultured in an incubator at 37°C and 5% CO_2_. Corresponding adenovirus vectors were transfected for different experiments to study the role of p16^INK4a^ in CMs.

### 2.6. Recombinant Adenovirus

Recombinant adenovirus of p16^INK4a^ (Ad5:cTNT-INK4a), p16^INK4a^ scrambled shRNA (Ad5:cTNT-INK4a RNAi), and adenovirus of control (Ad5:cTNT-CON) were designed by CMs specific cTNT promoter obtained from GeneChem Company (China). Ad5:cTNT-CON served as an empty control virus for Ad5:cTNT-INK4a and Ad5:cTNT-INK4a RNAi. It was found in previous studies of primary CMs in vitro that we used a multiplicity of infection (MOI) = 50 as a viral titer to transfect CMs 48 hours after performing other relevant experimental assays [17, 21]. In vivo, 1-day-old neonatal mice underwent AR + intramyocardial injection: Ad5:cTNT-INK4a RNAi (3.0 × 10^7^ PFU/mouse, total volume = 6 *μ*l/mouse) or Ad5-cTNT-CON (3.0 × 10^7^ PFU/mouse, total volume = 6 *μ*l/mouse).

### 2.7. Treatment of N-Acetylcysteine (NAC), Palbociclib (PAL), and Rapamycin (RPM) In Vitro

NAC is widely used in research as a scavenger of ROS. According to the grouping, pretreated CMs were further treated with 2.5 *μ*M NAC for 2 h or not to achieve the intervention on ROS. PAL is widely used as a CDK4 and Cdk6 inhibitor in clinical therapy and basic research. Pretreated CMs were further treated with 0.5 *μ*M PAL which has achieved an inhibitory effect on CDK4/6; also, pretreated CMs were further treated with the RPM (GlpBio, GC15031) at 10 nM for 24 h to attain the induction of autophagy.

### 2.8. Reverse Transcription and Real-Time Fluorescence Quantitative Polymerase Chain Reaction (qPCR)

Tissues or cells were crushed with Trizol (Thermo Fisher Scientific) and placed in enzyme-free EP tubes, which were left to rest for 5 minutes. Each tube was added with 200 *μ*l chloroform (Thermo Fisher Scientific), rested for 5 minutes after 30 seconds of severe vibration, and then placed in a new 1.5 ml EP tube. An equal volume of isopropyl alcohol was added, mixed, and left to rest for 10 minutes at room temperature. After centrifugation for 10 minutes, white precipitates of RNA were found at the bottom of the tube. The supernatant was discarded, dried with filter paper, and dried at room temperature for 5-10 minutes. 20 ml of DEPC water was added to each tube to dissolve the RNA. The concentration and purity of OD260/OD280 were measured by Nanodrop 2000 spectrophotometer (Thermo Fisher Scientific). Two-step real-time PCR (real-time PCR) was used to detect the expression levels of related RNA in each group. Real-time PCR was performed in Roche LightCycler 96 after reverse transcription to detect relevant molecular RNA expression levels using specific primers and SYBR Green (Vazyme Biotech, Nanjing, China; Q131-02). The relative expression of target genes was normalised to the 18S. The sequences of qRT-PCR primers were as follows: 18S-F: TTTCTTAGCGGGAGCGT, 18S-R: GCTGCTTCTGAGGTTTGG; INK4a-F: GCTTCTCACCTCGCTTGTC, INK4a-R: CGCTGCTGTACTCCCTCA.

### 2.9. Cell Cycle Analysis by Flow Cytometry

To observe the effect of p16^INK4a^ on the cell cycle, CMs were treated with Ad5-cTNT-INK4a or Ad5-cTNT-INK4a RNAi for 48 hours, and control virus groups were established. CMs were digested with 0.25% trypsin without EDTA and centrifuged (1200 rpm, 5 min, 4°C). Then, the cells were washed twice using precooled PBS and resuspended with 70% ethanol. The ethanol suspension was stored overnight in a 4°C refrigerator for cell fixation. Finally, PINase staining buffer (BD Biosciences, USA) was used for dyeing and flow cytometry detection.

### 2.10. Immunofluorescence Staining

Fresh heart tissue was washed off in PBS and fixed in a 4% neutral formaldehyde solution for 24 h. After dehydrating and dipping wax, hearts were embedded with paraffin. The slices were made by paraffin slicer with a thickness of 5 *μ*m. The paraffin sections were dewaxed and rehydrated. The rehydrated sections were placed in an acid repair solution in a microwave oven for 3 min at high and 7 min at low temperatures. After recovering to room temperature, a 5% BSA blocking solution was used to block for 2 hours in the wet box at room temperature. Ki67, phosphorylated histone 3 (pH 3), AuroraB antibody, and the Click-iT EdU imaging Kits (Thermo Fisher) were used to culture to identify cell cycle activity. CMs were labelled with cardiac troponin T (cTNT), and the nucleus was labelled with Hoechst or 6-diamidino-2-phenylindole (DAPI). Cell membranes were stained by wheat germ agglutinin (WGA) staining (Thermo Fisher; w32466). After glycerol sealing, the film was taken by a confocal laser. Quantitative data were obtained with confocal microscopy (Zeiss, Oberkochen, Germany) and Carl Zeiss microscopy (Germany). To detect CM proliferation, we calculated the numbers of EdU^+^, Ki67^+^, pH 3^+^, and AuroraB^+^ CMs across the infarct boundary zone and their proportions in all boundary CMs. The specific antibody information was as follows: anti-Ki67, Abcam, ab15580, 1 : 200; anti-pH 3, CST, 9706, 1 : 200; and anti-cTNT, Abcam, Ab8295, 1 : 200.

### 2.11. Western Blot (WB)

The protein was added to 6× Loading Buffer (SDS-PAGE protein Loading Buffer), boiled in a 95°C metal bath for 5-10 min, and then cooled to room temperature. According to the specification of SDS-PAGE adhesive, a suitable concentration of separation adhesive was prepared. The procedures were as follows: marker 3-5*μ*l, protein 20-30*μ*g, electrophoresis: the concentration gel was used for 20-25 min, and the separation gel was used for 60-90 min, the preparation of PVDF membrane (Millipore): the length of PVDF membrane was 9 cm × the width of 6 cm, and it was immersed in methanol for 5 min; rotating film: 300 mA constant current for 2 h, or 90 V constant pressure for 2 h, sealing: 5% BSA or 5% skim milk powder (all with TBS configuration), shaking bed at room temperature stuffing for 2 h; antibody incubation: the first antibody diluent was added to 4°C of horizontal shaking machine and incubated overnight, TBST was washed for 30 min × 3 times, then the second antibody diluent was added to the horizontal shaking machine and incubated for 2 h at room temperature, and TBST was washed for 30 min × 3 times. ECL developer was prepared in a 1 : 1 ratio. The band intensity was quantified using Image J software (National Institutes of Health, United States). The specific antibody information was as follows: anti-p16INK4, Proteintech, 10883-1-AP, 1 : 1000; anti-CDK4, Proteintech, 11026-1-AP, 1 : 1000; anti-CDK6, Proteintech, 19117-1-AP, 1 : 1000; anti-Beclin1, Proteintech, 11306-1-AP, 1 : 1000; anti-ATG5, Proteintech, 10181-2-AP, 1 : 1000; anti-LC3B, Proteintech, 18725-1-AP, 1 : 1000; anti-CyclinD1, Proteintech, 26939-1-AP, 1 : 1000; anti-GAPDH, CST, 5174, 1 : 1000; and anti-LaminB1, Abcam, ab16048, 1 : 1000.

### 2.12. Mass Spectrometry and LC-MS/MS Analytical Processing

The preparation methods of all samples used for quantitative proteomics refer to the standard procedure. CMs were extracted from neonatal 1-day-old mice and cultured for 24 h to observe cell adherence. The cells were transfected with Ad5-cTNT-INK4a RNAi and Ad5-cTNT-CON using MOI = 50 as the virus titer for 48 h after fluid change according to cell adherence and growth status. TMT six standard: (3 test vs. 3 control, 6 channels), each channel corresponds to one biological repeat, each biological repeat using 200 mM triethylammonium bicarbonate (TEAB) redissolved 750 *μ*g peptide powder. A total of 5 sets of TMT labelling reagents were used to label 6 groups (3 experimental + 3 control) samples.

### 2.13. Frozen Section and ROS Assay

Take fresh hearts and embed them in OCT of microtome freezer, slice it into 5 *μ*m thickness, and mark ROS with ROS Assay Kit following the standard protocol (Beyotime, China). The ROS in the myocardium can oxidise nonfluorescent DCFH to generate fluorescent 7′-dichlorofluorescein (DCF). The level of ROS in the myocardium can be expressed by detecting the fluorescence intensity of DCF. In vitro, NMCMs were extracted and treated with Ad5-cTNT-INK4a and Ad5-cTNT-CON for 48 h. Mark ROS with ROS Assay Kit following the standard protocol (Beyotime, China). The ROS in the myocardium can oxidise nonfluorescent DCFH to generate fluorescent DCF. The level of ROS in the myocardium can be expressed by detecting the fluorescence intensity of DCF. The fluorescence intensity of DCF is analysed by Image J.

### 2.14. Measurement of Fluorescent LC3 Puncta

A double-dot fluorescence overlay-orange fluorescence protein tandem labelled LC3 expression method was used to determine the LC3 fluorescence dot sites after the transduction of mRFP-GFP-LC3 in CMs (HanBio Technology, China; MOI = 50) according to the manufacturer's instructions. After various treatments, the CMs were rinsed with PBS, fixed with 4% paraformaldehyde, and observed under the Zeiss Cell Observer confocal laser scanning microscope 710 (Zeiss, Germany). The number of mRFP and GFP spots was measured manually by counting fluorescence spots. The red spots are the autophagolysosomes (mRFP), and the yellow spots are the autophagosomes (mRFP+GFP).

### 2.15. Transmission Electron Microscopy

The hearts were cut into 1 mm slices and fixed overnight at 4°C in 4% glutaraldehyde and 1% osmic acid. Then, the samples were sent to the Electron Microscope Center of Nanjing Medical University for further standardised preparation. The myocardial ultrastructure was examined by transmission electron microscopy (TEM) (JEOL JEM-1400Flash TEM system).

### 2.16. Statistical Analysis

Experimental data were analysed with SPSS 22.0 (IBM, United States), and results are presented as ^“^Mean ± SEM^”^. Differences between the two groups were compared using Student's *t*-test. Multiple groups in each specific experiment design shown in the figure were compared using one-way ANOVA with post hoc test using Tukey's test and multiple-way ANOVA with post hoc test using Bonferroni's test, respectively. The results with *P* values < 0.05 were considered statistically significant (^∗^ indicating *P* < 0.05, ^∗∗^ indicating *P* < 0.01, ^∗∗∗^ indicating *P* < 0.001). All graphs and tables are drawn using GraphPad 8.0.

## 3. Results

### 3.1. The Expression of p16^INK4a^ Is Related to the Myocardial Regeneration Time Window

**Figure 1:**
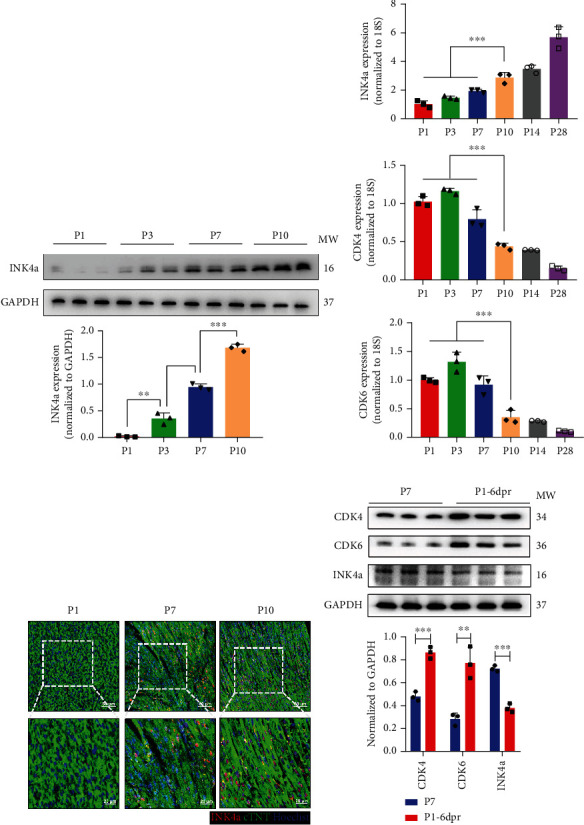
The expression of p16^INK4a^ is related to the myocardial regeneration time window. (a) Western blot analysis of p16^INK4a^ protein expression in mice myocardium at P1, P3, P7, and P10. (b) Changes of mRNA levels of p16^INK4a^, CDK6, and CDK4 in mice myocardium of P1 to P28 (*n* = 3). (c) The expression of p16^INK4a^ in the hearts of P1, P7, and P10 mice was detected by immunofluorescence staining. Scale bar: 20 *μ*m, 50 *μ*m. (d) The expression of p16^INK4a^, CDK6, and CDK4 in P7 and P1-6dpr neonatal hearts was detected by Western blot (data are presented as mean ± SEM, ^∗∗^*P* ≤ 0.01, ^∗∗∗^*P* ≤ 0.001).

There is a relatively short period for the regenerative repair of neonatal mammalian myocardial tissue. The regeneration time window of newborn mice myocardium is about 7 days, and the regeneration ability gradually decreases during these 7 days [3]. To explore the relationship between p16^INK4a^ and myocardial regeneration, we focused on expression patterns of p16^INK4a^ during perimyocardial regeneration. WB results showed that p16^INK4a^ expression gradually increased after birth, which was consistent with the timeline of the decline of myocardial regeneration ability (Figure 1(a)). Subsequently, we found that the p16^INK4a^ mRNA level significantly differed between the regeneration time and afterwards period by q-PCR, consistent with the downward trend of regeneration capacity. In addition, as a classical regulatory regeneration pathway of p16^INK4a^, CDK4/6 also showed a reverse trend with p16^INK4a^ during myocardial regeneration (Figure 1(b)). In vivo, we detected the expression of p16^INK4a^ by immunofluorescence staining. Similarly, the results showed that p16^INK4a^ expression gradually increased with the loss of myocardial regeneration ability and enriched in the nucleus (Figure 1(c)). In addition, we constructed AR in neonatal mice at P1 to induce myocardial regeneration, and the results showed that p16^INK4a^ expression decreased while CDK4/6 expression increased during cardiac regenerative repair (Figure 1(d)). The above results indicated that p16^INK4a^ expression was negatively correlated with the change in myocardial regenerative repair ability.

### 3.2. P16^INK4a^ Regulates the Proliferation of NMCMs In Vitro

**Figure 2:**
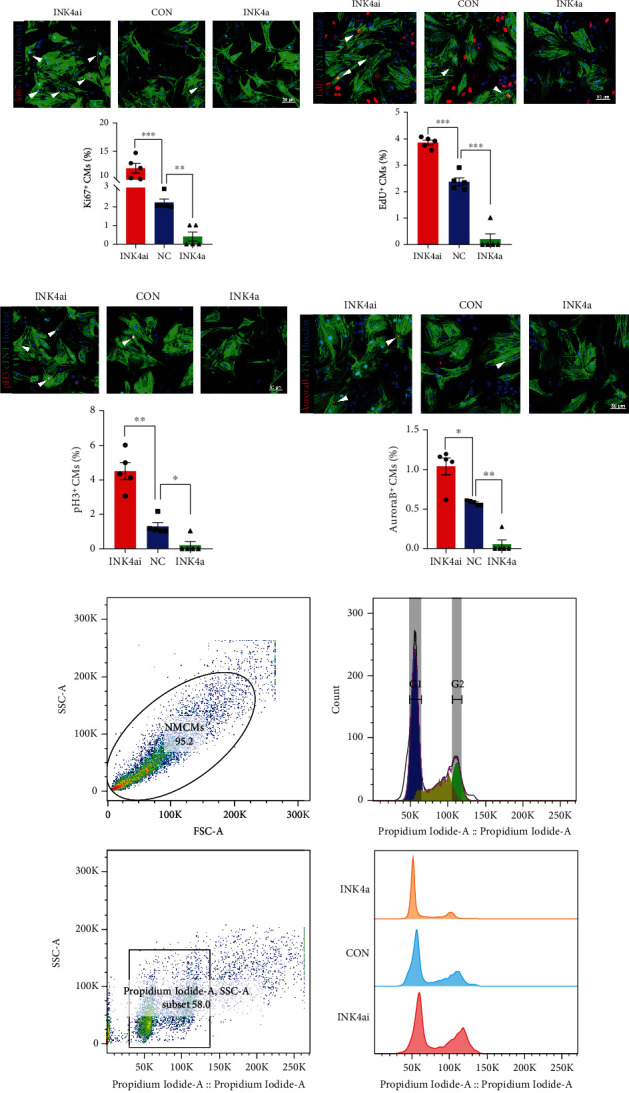
P16^INK4a^ regulates the proliferation of NMCMs in vitro. (a, b) Cardiomyocytes were transfected with Ad5:cTnT-CON (NC), Ad5:cTnT-INK4a (INK4a), or Ad5:cTnT-INK4a RNAi (INK4ai), and p16^INK4a^ expression was detected by Western blot and qRT-PCR (*n* = 3). (c–f) Cardiomyocyte proliferation is quantified by immunofluorescence for Ki67, pH 3, EdU, and Aurora B in cardiomyocytes transfected with INK4ai/NC/INK4a (*n* = 5). Scale bar: 50 *μ*m. (g) The G0/1, S, and G2/M phase proportions of INK4ai/NC/INK4a cardiomyocytes were detected by flow cytometry and cell cycle analysis (*n* = 3) (data are presented as mean ± SEM; NS: no statistical difference, ^∗^*P* ≤ 0.05, ^∗∗^*P* ≤ 0.01, ^∗∗∗^*P* ≤ 0.001).

To investigate the relationship between p16^INK4a^ and myocardial regeneration, we extracted and purified NMCMs and constructed adenovirus vectors carrying CM-targeted p16^INK4a^ overexpression or knockdown. We first verified the transfection efficiency by WB and q-PCR (MOI = 50). The results showed that transfection of Ad5:cTNT-INK4a (INK4a) could achieve the overexpression of p16^INK4a^ at the protein and mRNA levels; transfection of Ad5:cTNT-INK4a RNAi (INK4ai) can significantly downregulate p16^INK4a^ (Figures 2(a) and 2(b)). Then, we explored the effect of p16^INK4a^ on the proliferation of NMCMs by immunofluorescence staining of proliferation indicators, such as EdU (DNA synthesis), Ki67 (cell cycle activity), pH 3 (mitosis), and Aurora B (mitosis spindle). The results showed that p16^INK4a^ knockdown could significantly improve the proliferation of NMCMs, while overexpression of p16^INK4a^ could significantly inhibit the proliferation ability of NMCMs (Figures 2(c)–2(f)). In addition, PI staining and flow cytometry results indicated that the proportion of G0/1 phase was significantly decreased and the ratio of S phase or G2/M phase was significantly increased after p16^INK4a^ knockdown, while the function of overexpression p16^INK4a^ was contrary (Figure 2(g)). The results showed that p16^INK4a^ could substantially affect the activation of cell proliferation signals and regulate the progression of the cell cycle.

### 3.3. Knockdown p16^INK4a^ Prolongs the Period of Myocardial Regeneration in Neonatal Mice In Vivo

**Figure 3:**
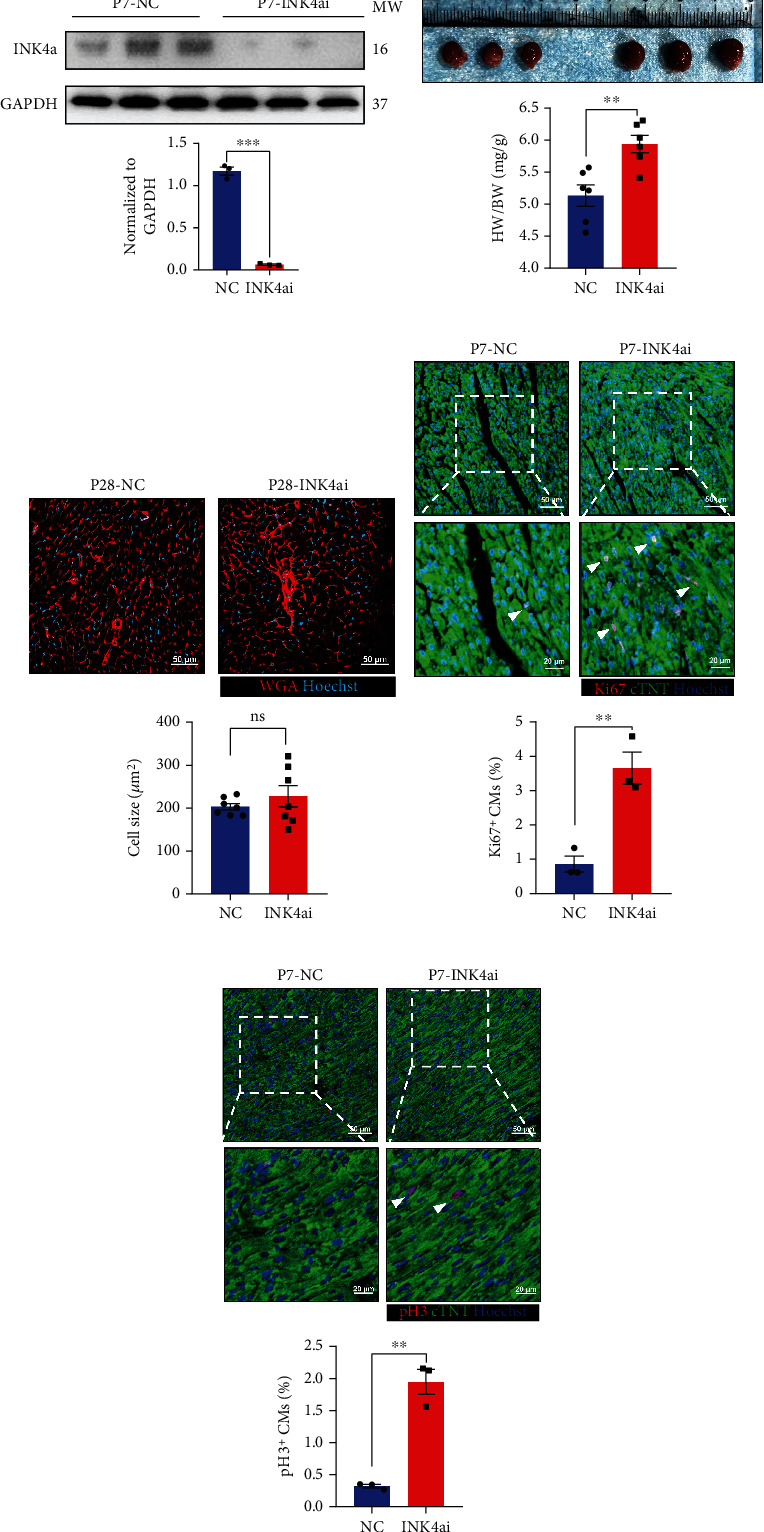
Knockdown p16^INK4a^ prolongs the period of myocardial regeneration in neonatal mice in vivo. (a) Western blot analysis was performed to detect the expression of p16^INK4a^ in the myocardium at P7 after intraperitoneal injection of Ad5:cTnT-CON (NC) or Ad5:cTnT-INK4a RNAi (INK4ai). (b) Cardiac appearance and the ratio of heart weight and body weight (HW/BW) in mice at P28 after intraperitoneal injection of NC or INK4ai (*n* = 6). (c) WGA staining was used to count the cardiomyocyte size changes at P28 after intraperitoneal injection of NC or INK4ai (*n* = 7). Scale bar: 50 *μ*m. (d, e) The proliferation proportion of cardiomyocytes in the heart at P7 was counted by Ki67 and pH 3 immunofluorescence after intraperitoneal injection of NC or INK4ai in neonatal mice (*n* = 3). Scale bar: 20 *μ*m, 50 *μ*m (data are presented as mean ± SEM; NS: no statistical difference, ^∗∗^*P* ≤ 0.01, ^∗∗∗^*P* ≤ 0.001).

As mentioned above, we have found that p16^INK4a^ could interfere with the proliferation cycle of CMs in vitro. Next, we explored the effects of p16^INK4a^ on the neonatal mice myocardium under physiological conditions in a diverse and complex environment. We injected Ad5:cTNT-INK4a RNAi (INK4ai) and Ad5:cTNT-CON (NC) intraperitoneally in P1 mice. Hearts were harvested at 7 days postnatal (P7), and the successful intervention of adenovirus was verified by WB (Figure 3(a)). Compared with the NC group, the volume and weight of the heart in the INK4ai group were significantly increased at P28 (Figure 3(b)). To determine whether the knockdown of p16^INK4a^ can affect the whole heart by increasing the size or the number of CMs, we used WGA staining and statistical analysis of cell size in vivo. It was found that the knockdown of p16^INK4a^ could lead to heterogeneous hypertrophy of CMs, but there was no statistical difference (Figure 3(c)). Subsequently, the cell proliferation indicators were detected by immunofluorescence staining in vivo. The results showed that the proportion of Ki67 and pH 3 positive CMs in the INK4ai group was significantly higher than that in the NC group at P7 (Figures 3(d) and 3(e)). The above results indicated that at the time point (P7) when the CM proliferative ability was weakened, inhibiting p16^INK4a^ could comparatively prolong the proliferative period of neonatal myocardium.

### 3.4. Overexpression p16^INK4a^ Inhibits Myocardial Regeneration and Repair after AR in Neonatal Mice

**Figure 4:**
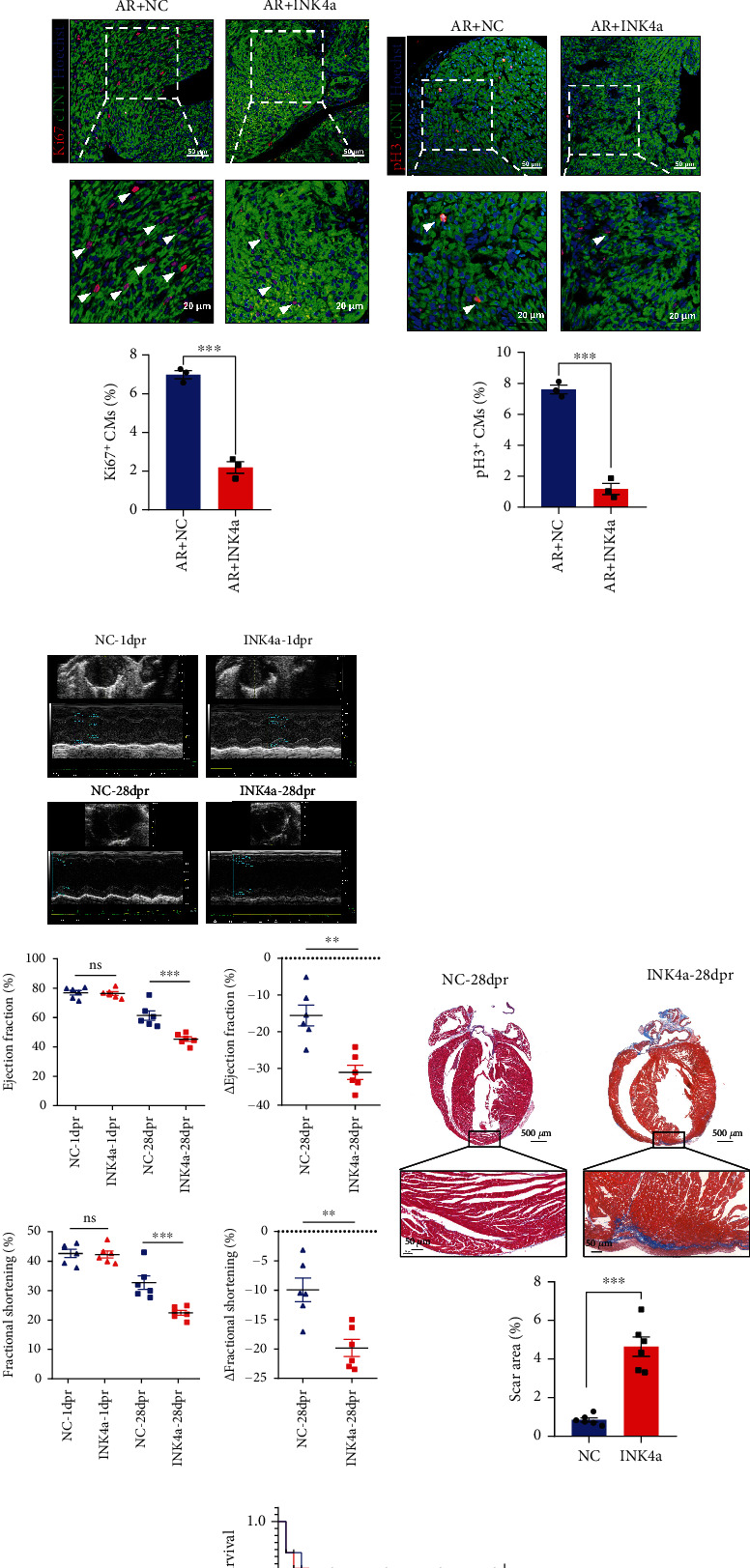
Overexpression p16^INK4a^ inhibits myocardial regeneration and repair after AR in neonatal mice. (a, b) Western blot and qRT-PCR were used to detect the expression of p16^INK4a^ in ventricular muscle after specific overexpression of p16^INK4a^ at 6 dpr (*n* = 3). (c, d) The proliferation proportion of cardiomyocytes in the heart at 6 dpr was counted by Ki67 and pH 3 immunofluorescence after myocardial injection of NC or INK4a in neonatal mice (*n* = 3). Scale bar: 20 *μ*m, 50 *μ*m. (e) Cardiac function was detected by echocardiography at 1 dpr and 28 dpr after AR (*n* = 6). (f) Comparison of scar size by Masson staining between the NC and INK4a groups at 28 days after AR (*n* = 6). Scale bar: 50 *μ*m, 500 *μ*m. (g) Overall survival rate of neonatal mice in the P1-P28 stage after myocardial injection in situ of NC or INK4a after AR (*n* = 10) (data are presented as mean ± SEM; NS: no statistical difference, ^∗^*P* < 0.05, ^∗∗^*P* < 0.01, ^∗∗∗^*P* < 0.001).

To further explore the effect of p16^INK4a^ on the regenerative repair of neonatal myocardium in vivo, we performed AR surgery on P1 mice and injected Ad5:cTNT-INK4a or Ad5:cTNT-CON into the myocardium. WB and q-PCR results showed that the expression of p16^INK4a^ in the AR + INK4a group was significantly increased than in the AR + NC group at 6 days postresection (6 dpr) (Figures 4(a) and 4(b)). Subsequently, compared with the AR + NC group, the proportion of Ki67 and pH 3 proliferative staining was significantly reduced in the AR + INK4a group (Figures 4(c) and 4(d)). To explore the effect of p16^INK4a^ on cardiac function, we performed echocardiography at 1 dpr and 28 dpr. The result of echocardiography showed no significant difference in cardiac function between the two groups, indicating that AR caused equivalent myocardial damage in both groups at 1 dpr (EF: AR + NC: 76.92 ± 1.39%, AR + INK4a: 76.37 ± 1.25%; FS: AR + NC: 42.67 ± 1.27%, AR + INK4a: 42.29 ± 1.20%; *P* > 0.05, *n* = 6). However, the result of echocardiography showed that the indexes related to cardiac contractile function decreased significantly in the AR + INK4a group compared with the AR + NC group at 28 dpr (EF: AR + NC: 61.35 ± 3.20%, AR + INK4a: 45.31 ± 1.53%; FS: AR + NC: 32.72 ± 2.30%, AR + INK4a: 22.46 ± 0.86%; *P* < 0.001, *n* = 6) (Figure 4(e)). Additionally, there was a significant myocardial injury scar at 28 dpr in the AR + INK4a group via Masson staining, indicating overexpression p16^INK4a^ inhibited the structural recovery of neonatal myocardium (Figure 4(f)). The overall survival rate was similar between the AR + NC and AR + INK4a groups (Figure 4(g)). The above results indicated that overexpression of p16^INK4a^ could hinder the functional and structural recovery of the neonatal heart.

### 3.5. Proteomics and Bioinformatics Analysis

**Figure 5:**
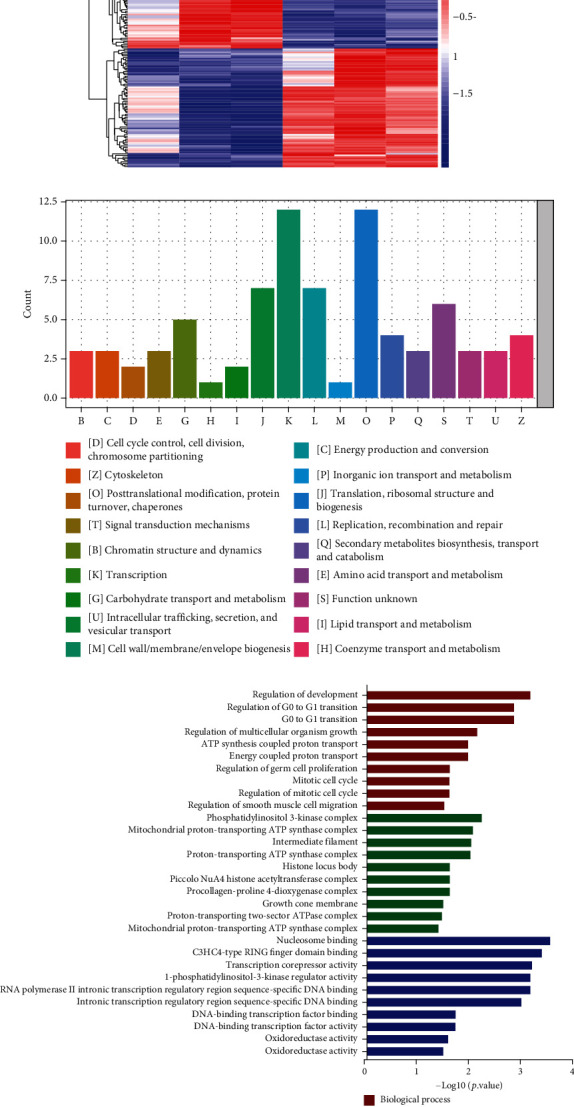
Proteomics analysis shows that knockdown P16 activates cell cycle processes and energy metabolism balance. (a) Volcano plot of upregulated, downregulated, and unchanged proteins in the Ad5:cTnT-INK4a RNAi (INK4ai) group versus the Ad5: cTNT-CON (NC) group; multiples change greater than 1.5 times and *P* value < 0.05. (b) Heatmap of cluster analysis of differentially expressed proteins among cardiomyocytes treated with INK4ai or NC. (c) Histograms of the frequency of KOG functional classifications, with the abscissa representing the functional annotation of each bar classification, indicated by an alphabetical abbreviation; the ordinate indicates the number of proteins corresponding to each classification. (d) GO secondary classification histograms; the ordinate indicates the enriched GO functional classification into BP, CC, and MF. The abscissa represents the -log10 (*P* value) value corresponding to each entry. (e) KEGG functional enrichment bubble plot; the colour gradient represents the size of the *P* value, and the bubble size represents the number of differentially expressed proteins involved in this KEGG pathway.

To explore the relevant mechanism of promoting CM proliferation by P16 knockdown, we transfected NMCMs with Ad5:cTNT-INK4ai (INK4ai) and Ad5:cTNT-NC (NC). Then, we performed the quantitative proteomic analysis with TMT labelling between the two groups. A total of 89502 peptides and 9614 proteins were identified, among which 8903 proteins could be quantified. The differential expression multiple greater than 1.5 times and *P* value < 0.05 were used as the change threshold of significant difference for screening. The differentially expressed proteins were displayed in the form of a volcano plot. Compared with the NC group, 75 proteins were upregulated, and 76 were downregulated in the INK4ai group; detailed results were shown in the supplementary material (Figure 5(a), Table S1). The hierarchical cluster algorithm was adopted to analyse the significantly differentially expressed proteins in each comparison group, and heatmap data were displayed (Figure 5(b)). We undertook Clusters of Orthologous Groups of proteins (COG) analysis on the differential proteins and used KOG, the eukaryotic-specific version of COG, for functional annotation. The results showed that the cell behaviour changed in the process of cell cycle regulation, energy metabolism balance, and transcription translation after p16^INK4a^ knockdown in NMCMs (Figure 5(c)). The differentially expressed proteins were further annotated with the Gene Ontology (GO). We conducted significant enrichment analysis on differentially expressed proteins in GO secondary annotation. The results showed that differentially expressed proteins were enriched in cell cycle regulation, ATP energy metabolism, cell development, and other related GO entries (Figure 5(d)). The Kyoto Encyclopedia of Genes and Genomes (KEGG) was one of the databases commonly used for pathway research. By analysing and calculating the significance level of protein enrichment of each pathway, we found that the differential protein was enriched in JAK-STAT and mTOR signalling pathways (Figure 5(e)). And these signal pathways were closely related to cell cycle regulation and ROS metabolism in previous studies [22–25]. These results suggested that the knockdown of p16^INK4a^ may promote CM proliferation by regulating cell cycle progression and ROS metabolism.

### 3.6. P16^INK4a^ Mediates Abnormal Accumulation of ROS and Autophagy

**Figure 6:**
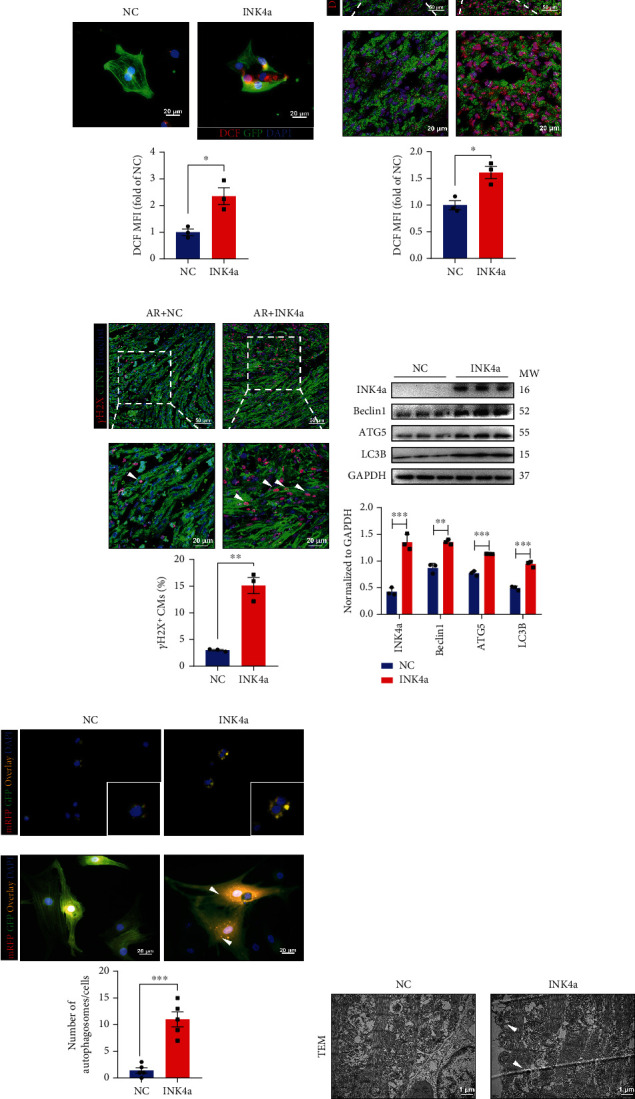
P16^INK4a^ mediates abnormal accumulation of ROS and autophagy. (a) The mean fluorescence intensity of DCF in cardiomyocytes transfected with Ad5:cTnT-INK4a (INK4a) or Ad5:cTnT-CON (NC) was determined by immunofluorescence *in vitro* (*n* = 3). Scale bar: 20 *μ*m. (b) The mean fluorescence intensity of DCF in cardiomyocytes transfected with INK4a or NC was determined by immunofluorescence at 6 dpr (*n* = 3). Scale bar: 20 *μ*m, 50 *μ*m. (c) DNA damage is quantified by immunofluorescence for the staining of *γ*H2X in cardiomyocytes at 6 dpr (*n* = 3). Scale bar: 20 *μ*m, 50 *μ*m. (d) The expression of p16^INK4a^, Beclin1, ATG5, and LC3B in the ventricular muscle after specific overexpression of p16^INK4a^ in 6dpr. (e) The number of autophagosomes was statistically analysed by immunofluorescence staining in INK4a or NC group (*n* = 5). Scale bar: 20 *μ*m. (f) The effect of p16^INK4a^ overexpression on autophagosomes of mice cardiomyocytes during 28 dpr was detected by transmission electron microscopy (*n* = 3) (data are presented as mean ± SEM, ^∗^*P* ≤ 0.05, ^∗∗^*P* ≤ 0.01, ^∗∗∗^*P* ≤ 0.001).

The relationship between p16^INK4a^ and oxidative stress is highly dependent on the environment and the energy utilisation of the cellular system. Thus, it appears that p16^INK4a^ is bidirectionally and interactively regulated by the presence of ROS in previous research [26–28]. To determine the relationship between p16^INK4a^ and ROS in CMs retaining proliferation ability, we labelled ROS in NMCMs by DCF. We found that the mean fluorescent intensity (MFI) of DCF in the INK4a group was significantly higher than in the NC group, indicating overexpression of p16^INK4a^ could lead to ROS accumulation (Figure 6(a)). Similarly, we also detected DCF and found extensive ROS accumulation in the AR + INK4a group at 6 dpr, with a wide distribution in CMs (Figure 6(b)). The accumulation of ROS could lead to DNA damage, which results in the withdrawal of the cell cycle and loss of division and proliferation ability. Therefore, the results showed that *γ*H2X, the DNA damage marker, increased significantly in the AR + INK4a group at 6 dpr, indicating overexpression p16^INK4a^ could cause more significant DNA damage (Figure 6(c)). Considering the change of intracellular ROS level was involved in the regulation of the whole autophagic flow process, and the bioinformatics analysis showed that the enriched JAK-STAT and mTOR signal pathways were widely involved in the occurrence and development of autophagy. Therefore, whether the heterogeneous expression of p16^INK4a^ could regulate autophagy in response to different levels of ROS is worth further investigation. The WB assay showed that the expression of autophagy indicators Beclin1, ATG5, and LC3B significantly increased in the AR + INK4a group, indicating p16^INK4a^ initiated significant autophagy activity (Figure 6(d)). Subsequently, NMCMs were transfected with mRFP-GFP-LC3 autophagy adenovirus, and autophagosome formation was detected by immunofluorescence. Compared with the NC group, the number of autophagosomes was significantly increased in the INK4a group (Figure 6(e)). In addition, more autophagosomes were also found in myocardial tissue through TEM after p16^INK4a^ overexpression in vivo (Figure 6(f)). These above results indicated that p16^INK4a^ caused abnormal accumulation of ROS and autophagy in CMs.

### 3.7. Alleviation of ROS by NAC Relieves P16-Mediated CDK4/6 Regulation of NMCM Proliferation

**Figure 7:**
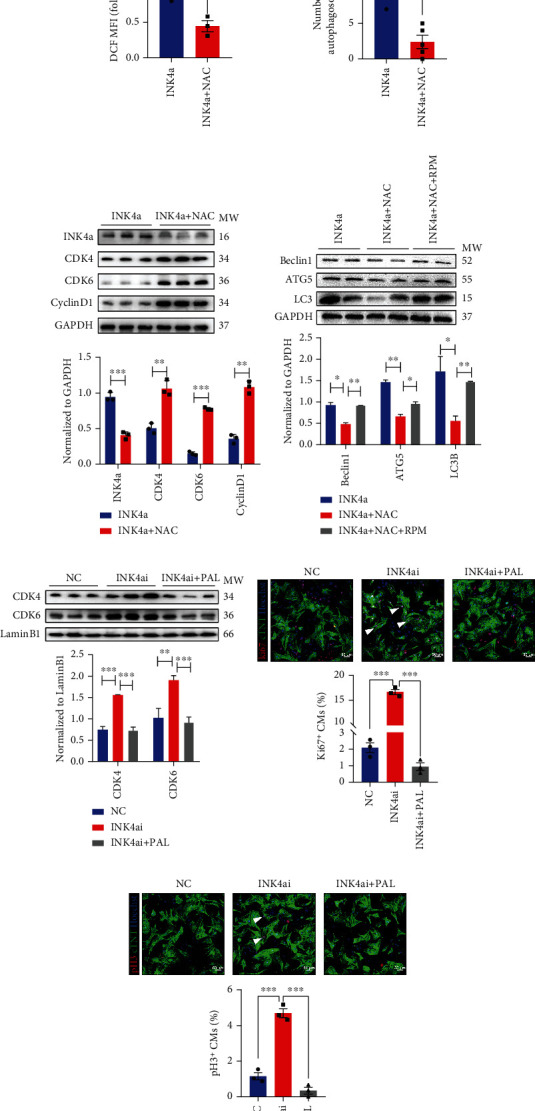
Alleviation of ROS by NAC relieves p16^INK4a^-mediated CDK4/6 regulation of NMCM proliferation. (a) The mean fluorescence intensity of DCF in cardiomyocytes treated with Ad5:cTnT-INK4a (INK4a) or INK4a + NAC was determined by immunofluorescence (*n* = 3). Scale bar: 20 *μ*m. (b) The number of autophagosomes was statistically analysed by immunofluorescence staining in INK4a or INK4a + NAC group (*n* = 5). Scale bar: 20 *μ*m. (c) The expression of p16^INK4a^, CDK4/6, and CyclinD1 in cardiomyocytes treated with INK4a or INK4a + NAC was detected by Western blot. (d) The expression of Beclin1, ATG5, and LC3B in cardiomyocytes treated with INK4a, INK4a + NAC, or INK4a + NAC + RPM was detected by Western blot. (e) The expression of CDK4 and CDK6 in cardiomyocytes treated with NC, Ad5:cTnT-INK4ai (INK4ai), or INK4ai + PAL was detected by Western blot. (f, g) Cardiomyocyte proliferation is quantified by immunofluorescence for Ki67 and pH 3 in cardiomyocytes transfected with NC/INK4ai/INK4ai + PAL (*n* = 3). Scale bar: 50 *μ*m (data are presented as mean ± SEM, ^∗^*P* ≤ 0.05, ^∗∗^*P* ≤ 0.01, ^∗∗∗^*P* ≤ 0.001).

Our above results suggested that the antiproliferative effect, ROS level, and autophagy regulation produced by p16^INK4a^ could be related or concomitant. Subsequently, NAC was used together with Ad5:cTNT-INK4a in NMCMs. The detection of DCF-labeled ROS showed that NAC could significantly reduce ROS increase caused by overexpression p16^INK4a^ (Figure 7(a)). Concurrently, it reduced autophagy caused by ROS increase (Figure 7(b)). In addition, we detected the targets of p16^INK4a^ regulating cell cycle (CDK4, CDK6) and the expression of CyclinD1 by WB assay. The results showed that NAC inhibited the expression of p16^INK4a^ and promoted the expression of CDK4/6 and CyclinD1. Therefore, NAC could regulate p16^INK4a^, CDK4/6, and CyclinD1 in a covering manner (Figure 7(c)). To further confirm the relationship between ROS, p16^INK4a^, and autophagy, we used NAC and autophagy inducer (RPM) to carry out a combination experiment on NMCMs overexpressing p16^INK4a^. Through WB detection, we found that ROS clearance can significantly inhibit the promotion of p16^INK4a^ on the expression of autophagy-related genes, and using an autophagy inducer could reverse this process (Figure 7(d)). As an inhibitor of CDK4/6, PAL is widely used in targeted cancer therapy to induce G0/G1 cell cycle arrest [29]. In our research, we further explored whether inhibition of p16^INK4a^ on the promotion of NMCM proliferation could be blocked by inhibiting CDK4/6 activity. We conducted a combined experiment of knockdown p16^INK4a^ and PAL. The WB assay showed that knockdown p16^INK4a^ could increase the nuclear components of CDK4 and CDK6 to exert positive regulation on the cell cycle. However, the use of PAL interfered with the regulatory effect of p16^INK4a^ on CDK4/6 (Figure 7(e)). Moreover, we found that the proliferation effect of activating NMCMs by inhibiting p16^INK4a^ was significantly blocked by PAL (Figures 7(f) and 7(g)). These results suggested that p16^INK4a^ regulated the proliferative ability through CDK4/6.

### 3.8. Research Pattern Diagram

**Figure 8:**
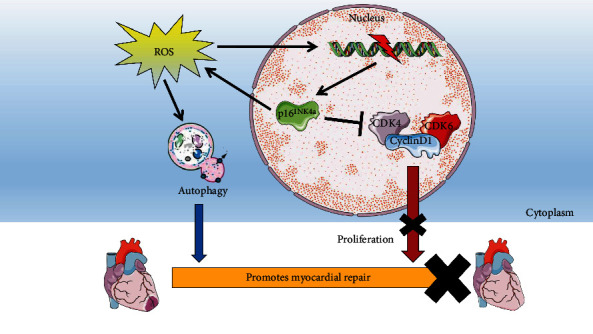
Research pattern diagram. P16^INK4a^ can not only regulate ROS metabolism to induce autophagy but also can affect the proliferation of cardiomyocytes by inhibiting CDK4/6, ultimately affecting the myocardial regeneration and repair after injury.

Previous studies have found that ROS stimulates DNA damage to increase p16^INK4a^. Our research found that overexpression p16^INK4a^ could reverse-regulate ROS metabolism to induce autophagy and inhibit CDK4/6, thus affecting CM proliferation. Finally, it inhibits regenerative repair after myocardial injury. Inhibition of p16^INK4a^ could promote myocardial regenerative repair in the above ways (Figure 8).

## 4. Discussion

This study demonstrated that p16^INK4a^ promoted CM proliferation and myocardial regeneration after injury in vitro and in vivo. Overexpression of p16^INK4a^ led to repair defects in structure and function in neonatal hearts. In contrast, the knockdown of p16^INK4a^ could improve the proliferation ability of CMs. Additionally, we found that p16^INK4a^ exerted its function through two pathways: p16^INK4a^ regulated CM cycle progression through CDK4/6 and ROS-mediated autophagy. In previous research, the specific roles of p21^WAF1^ and p27^KIP1^ have been clarified in regulating CM proliferation. The cell cycle arrest in CM is associated with the loss of CDK expression and the accompanying increase of cell cycle inhibitors p21^WAF1^ and p27^KIP1^, while p16^INK4a^ has not received much attention [11, 30]. Therefore, our research filled this gap by exploring p16^INK4a^ and the proliferation of CMs.

As a cardiovascular disease hotspot, p16^INK4a^ and p14^ARF^ are encoded by CDKN2a near the 9p21.3 genomic region [31]. P16^INK4a^ inhibits the phosphorylation of Rb mediated by CDK4/6, resulting in the release of E2Fs. And p14^ARF^ is the E2F target gene transcribed from the CDKN2a replacement reading frame, which response to excessive pRb phosphorylation and E2F activity by stabilising TP53 (the tumor protein p53) [31, 32]. P14^ARF^ had no homology in organisms with the adult CM regeneration ability (such as salamanders and zebrafish). Unlike p14^ARF^, p16^INK4a^ in adult CMs with proliferation is homologous [14]. Therefore, the research of p16^INK4a^ has more significance for mapping to explore the reactivation of mammalian CM proliferation.

In fact, on the one hand, many studies have shown that the redox state could program the process of cardiomyocyte proliferation and regeneration. In our research, as a ROS scavenger, NAC could significantly reduce ROS increase caused by overexpression p16^INK4a^ and regulate p16^INK4a^, CDK4/6, and CyclinD1 in a covering manner (Figures 7(a)–7(c)). On the other hand, p16^INK4a^ also has reverse regulation of ROS. It is reported that the overexpression of p16^INK4a^ will increase the ROS level in the human diploid fibroblast cell line (TIG-3). At the same time, the knockdown of p16^INK4a^ will reduce the ROS level in the conditional immortalised human fibroblast cell line (SVts8). The above indicates that the relationship between p16^INK4a^ level and oxidative stress may be highly dependent on the environment and cell system utilisation [26, 28, 33]. A thought-provoking question is whether p16^INK4a^ regulation of ROS is related to cell cycle regulation and whether p16^INK4a^ plays a regulatory role in these two interrelated processes. ROS is a by-product of the biological reaction of energy generation, which is mainly produced in mitochondria through oxidative metabolism. Under metabolic stress, intermediates such as glucose, glutamine, lactic acid, and fatty acid are alternately consumed to produce ATP, and the increased burden of energy metabolism increases the production of ROS. The conflict between generating energy and maintaining ROS homeostasis significantly affected cell fate [24]. In our results of proteomics and bioinformatics analysis, the change of p16^INK4a^ substantially affects the process of cell energy metabolism, which is closely related to cell cycle regulation and ROS metabolism (Figures 5(a)–5(e)). In addition, in the study of Lv et al., p16^INK4a^ regulates ROS production in HSC through p38/MAPK signal transduction rather than cell cycle dependence. Similarly, the role of p16^INK4a^ in regulating ROS levels during tumorigenesis is not significantly related to its role in cell cycle regulation. The adjusting of intracellular oxidative stress mediated by p16^INK4a^ is independent of the CDK4/6-Rb pathway rather than secondary to the potential cell cycle effect, thus confirming the new function of p16^INK4a^ [27]. Therefore, according to our research results, it seems reasonable that p16^INK4a^ may regulate myocardial regenerative repair through two interrelated pathways.

As terminally differentiated cells, the mammalian CMs enter a hyperoxic environment immediately after birth, and their metabolic mode changes from anaerobic glycolysis to mitochondrial oxidative phosphorylation [4, 34]. In oxidative phosphorylation, the electron transport chain causes the production and accumulation of ROS, which gradually increases with growth and development. This phenomenon is closely related to cellular senescence [35]. Excess ROS will cause DNA damage, which stops the cell cycle of CMs from proliferating, ultimately preventing the mammalian heart from regenerating repair after injury. Therefore, the timely elimination of ROS can have apparent effects on maintaining and prolonging the proliferation and regeneration ability of the myocardium [36]. In our research, p16^INK4a^ can reverse-regulate ROS metabolism and participate in cell cycle regulation as an influential factor of ROS-induced DNA damage, ultimately affecting myocardial regenerative repair after injury.

The results of the combination experiments between NAC and RPM on NMCMs overexpressing p16^INK4a^ indicated that ROS scavenging could significantly inhibit the promotion of p16^INK4a^ on the expression of autophagy-related genes, and the induction of autophagy occurred to reverse the process. It is reported that ROS and IRE1*α* can induce autophagy. Oxidative regulation of autophagy by ROS occurs in all stages of autophagy, from induction, phagocyte nucleation, phagocyte expansion, autophagic maturation, cargo transport to lysosomes, cargo degradation and product recovery, and the transcription of autophagy genes. Autophagy is an evolutionarily conserved recycling process in response to stressful conditions, including the production of ROS, which is a way of overactivated programmed cell death [37, 38]. At the same time, autophagy inhibition could prevent the clearance of dysfunctional mitochondria and lead to excessive ROS accumulation [39]. Our research found that p16^INK4a^ could activate autophagy, partly achieved by regulating ROS. We also found that overexpression of p16^INK4a^ overactivated autophagy, resulting in increased myocardial damage and weakened repair ability.

However, some limitations should be noted. In the CDKi family, whether other CDKi and INK4a play heterogeneous or synergistic functions in myocardial regenerative repair and related mechanisms, these pending issues need to be explored in the follow-up study. The work we have completed now shows that p16^INK4a^ could play an inhibitory role in myocardial regenerative repair beyond its known function as a cell cycle regulator, which seems to achieve dynamic fusion through the regulation of ROS. However, the specific mechanism of p16^INK4a^ regulating ROS is demonstrated in this manuscript through bioinformatics analysis and citing the research of others. In addition, although we have chosen to target the knockdown of p16^INK4a^ by CMs to enhance the ability of myocardial regenerative repair, given the inhibitory effect of p16^INK4a^ in the field of tumors, it is still worth further exploring whether there is ectopic potential tumorigenicity in the cardiac-specific knockdown of p16^INK4a^.

In summary, our research raised an attractive proposition that exits from the cell cycle, and autophagy state disturbances are indispensable for the loss of regenerative function of neonatal myocardium. The p16^INK4a^ would be a general mechanism to achieve this regulation. While it remains to be determined whether these alterations will contribute to the accompanying physiological transformation, the findings together highlight an exciting connection between the cell cycle, autophagy, and myocardial ageing. Given the critical role of p16^INK4a^ in cell proliferation, we found that the knockdown of p16^INK4a^ in promoting the proliferation of CMs provides an exciting perspective for understanding how cell proliferation harmonises with and contributes to changes occurring in cellular stress adaptation.

## 5. Conclusion

In summary, p16^INK4a^ regulated cardiomyocyte proliferation progression through CDK4/6 and ROS-related autophagy to jointly affect the myocardial regenerative repair. The present work expands our understanding of the senescence signal regulation in myocardial regeneration and indicates that targeting p16^INK4a^ might provide new therapeutic implications for ischemic heart disease.

## Data Availability

All data generated or analysed during this study are included in this article.

## Abbreviations

CMs: Cardiomyocytes
AR: Apical resection
ROS: Reactive oxygen species
CDKs: Cyclin-dependent kinases
Rb: RB1 protein
P1: 1 day postnatal
P7: 7 days postnatal
6 dpr: 6 days postresection
28 dpr: 28 days postresection
ICR: Institute of Cancer Research
MOI: Multiplicity of infection
EF: Ejection fraction
FS: Left ventricular fractional shortening
NAC: N-acetylcysteine
PAL: Palbociclib
RPM: Rapamycin
EDTA: Ethylenediaminetetraacetic acid
pH 3: Phosphorylated histone 3
WGA: Wheat germ agglutinin
EdU: 5-Ethynyl-2′-deoxyuridine
DAPI: 4′, 6-Diamidino-2-phenylindole
TEAB: Triethylammonium bicarbonate
DCFH: 2′,7′-Dichlorofluorescin diacetate
DCF: 7′-Dichlorofluorescein
TEM: Transmission electron microscopy
WB: Western blot
COG: Clusters of orthologous groups
GO: Gene Ontology
KEGG: Kyoto Encyclopedia of Genes and Genomes
MFI: Mean fluorescent intensity
SPF: Specific pathogen-free
cTNT: Cardiac troponin T
BSA: Bovine serum albumin.

## Supplementary Materials

Supplementary Materials Table 1: compared with the NC group, 75 proteins were upregulated, and 76 were downregulated in the INK4ai group; detailed results were shown below.
