# Supplementary material for: P16INK4a Regulates ROS-Related Autophagy and CDK4/6-Mediated Proliferation: A New Target of Myocardial Regeneration Therapy
Source: Oxid Med Cell Longev. 2023 Feb 18;2023:1696190. doi: 10.1155/2023/1696190 (PMC9966567; doi:10.1155/2023/1696190)
Supplement: Supplementary Materials — Table 1: compared with the NC group, 75 proteins were upregulated, and 76 were downregulated in the INK4ai group; detailed results were shown below. [file 1696190.f1.pdf]

protein	gene	gene_id	NC	INK4ai	Ratio	FC	log2FC
B7ZCT1	Rgs19	56470	53	80	1.52	1.52	0.60
Q922H7	Rasl11b	68939	241	403	1.67	1.67	0.74
B8JK87	Amotl2	56332	1,394	2,538	1.82	1.82	0.86
Q62225	Cish	12700	27	54	1.98	1.98	0.98
Q61696	Hspa1a	193740	4,792	9,261	1.93	1.93	0.95
Q9WTP2	Spry4	24066	259	143	0.55	-1.81	-0.86
P58500	Map3k7cl	224419	30	18	0.61	-1.64	-0.72
Q640N2	Arl13b	68146	32	66	2.05	2.05	1.04
P97467	Pam	18484	304	202	0.66	-1.51	-0.59
P62254	Ube2g1	67128	1,504	987	0.66	-1.52	-0.61
Q3UTC9	Mdm4	17248	69	111	1.59	1.59	0.67
Q04592	Pcsk5	18552	16	26	1.66	1.66	0.73
Q9CR42	Ankrd1	107765	3,111	4,729	1.52	1.52	0.60
Q91WC1	Pot1	101185	13	9	0.66	-1.51	-0.59
P63037	Dnaja1	15502	3,311	5,074	1.53	1.53	0.62
P98200	Atp8a2	50769	93	160	1.71	1.71	0.78
Q8BFW3	Ppp1r15b	108954	18	27	1.51	1.51	0.60
Q9CPU3	Tex46	67663	540	827	1.53	1.53	0.62
Q6ZWY8	Tmsb10	19240	431	1,976	4.59	4.59	2.20
A0A1L1SI	Oaf	102644	38	22	0.58	-1.72	-0.78
E9QAQ3	Arhgap26	71302	434	286	0.66	-1.52	-0.60
Q9EPW4	Clec3a	403395	232	141	0.61	-1.64	-0.72
A0A087W	Rnf2	19821	143	89	0.62	-1.61	-0.69
O54790	Mafg	17134	127	77	0.61	-1.65	-0.72
A0A2I3BF	Itih3	16426	641	1,037	1.62	1.62	0.69
E9Q7B0	P4ha1	18451	178	340	1.91	1.91	0.93
Q9WV27	Atp1a4	27222	151	100	0.66	-1.51	-0.60
Q8K273	Mmgt1	236792	190	125	0.66	-1.52	-0.60
A0A0U1R	Zfp646	233905	17	27	1.60	1.60	0.68
A8DIL0	Lgals8	56048	1,063	1,736	1.63	1.63	0.71
Q9DAM7	Tmem263	103266	41	24	0.59	-1.69	-0.76
P17183	Eno2	13807	3,921	2,608	0.67	-1.50	-0.59
Q9CQL1	Magohb	66441	1,490	2,254	1.51	1.51	0.60
E0CXE0	Grip2	243547	754	342	0.45	-2.21	-1.14
P50543	S100a11	20195	979	2,694	2.75	2.75	1.46
Q9Z1J3	Nfs1	18041	4,986	3,210	0.64	-1.55	-0.64
Q9DAV6	Serpinb9b	20706	1,420	2,243	1.58	1.58	0.66
Q8C142	Ldlrap1	100017	1,008	648	0.64	-1.55	-0.64
P18608	Hmgn1	15312	113	261	2.31	2.31	1.21
P48428	Tbca	21371	4,397	2,884	0.66	-1.52	-0.61
P03930	Mtatp8	17706	207	738	3.56	3.56	1.83
A0A087W	Aox3	71724	6	14	2.34	2.34	1.23
Q14CH1	Mocos	68591	28	17	0.62	-1.62	-0.69
A0A0A6Y	Clasp1	76707	15	80	5.30	5.30	2.41
Q9EP52	Twsg1	65960	368	240	0.65	-1.53	-0.62
Q3V1H1	Ckap2	80986	81	41	0.51	-1.97	-0.98

Q9Z2K1	Krt16	16666	235	102	0.44	-2.29	-1.20
Q6NT99	Dusp23	68440	67	40	0.59	-1.68	-0.75
Q8BG19	Tmtc4	70551	104	62	0.59	-1.69	-0.76
Q6P3Z4	Zfp37	22696	648	1,072	1.65	1.65	0.73
Q925J9	Med1	19014	774	1,183	1.53	1.53	0.61
Q99LW6	Yaf2	67057	61	93	1.53	1.53	0.61
Q9JJ48	Zc3h8	57432	97	169	1.74	1.74	0.80
Q9D032	Ssbp3	72475	8	19	2.41	2.41	1.27
Q9D1A0	Prxl2c	66129	117	185	1.58	1.58	0.66
A0A1L1SI	4930563M	75258	202	115	0.57	-1.77	-0.82
Q9WTR1	Trpv2	22368	250	377	1.51	1.51	0.59
Q80VY2	Inka2	109050	30	48	1.59	1.59	0.67
Q8R2L5	Mrps18c	68735	1,249	743	0.59	-1.68	-0.75
Q8BK30	Ndufv3	78330	914	343	0.38	-2.66	-1.41
Q9EPK2	Rp2	19889	302	158	0.52	-1.91	-0.93
Q9DB90	Smg9	71997	850	515	0.61	-1.65	-0.72
Q6P9N8	Trak2	70827	76	50	0.65	-1.53	-0.61
E9QA62	Lmod3	320502	537	294	0.55	-1.82	-0.87
Q91VA3	Capn8	170725	367	116	0.32	-3.16	-1.66
Q80TF4	Klhl13	67455	9	16	1.82	1.82	0.87
P06880	Gh1	14599	145	84	0.58	-1.74	-0.80
Q8R015	Bloc1s5	17828	152	96	0.63	-1.58	-0.66
E9Q8C1	Sgk1	20393	15	10	0.66	-1.50	-0.59
Q9ES52	Inpp5d	16331	85	56	0.66	-1.52	-0.60
Q9D1I6	Mrpl14	68463	838	1,434	1.71	1.71	0.77
E9PX57	Kif6	319991	387	258	0.67	-1.50	-0.59
A0A2I3BF	E4f1	13560	358	227	0.63	-1.58	-0.66
Q8VEN2	Plet1	76509	29	12	0.40	-2.48	-1.31
E9Q448	Tpm1	22003	387	256	0.66	-1.51	-0.59
B7ZNT7	Ankrd23	78321	149	266	1.78	1.78	0.83
P20065	Tmsb4x	19241	633	1,579	2.49	2.49	1.32
P60840	Ensa	56205	404	673	1.67	1.67	0.74
Q9CQX2	Cyb5b	66427	372	208	0.56	-1.79	-0.84
Q6P5E8	Dgkq	110524	82	51	0.62	-1.62	-0.70
Q3UDW8	Hgsnat	52120	247	148	0.60	-1.66	-0.74
Q921W2	Tial1	21843	28	18	0.66	-1.53	-0.61
Q6ZPL9	Ddx55	67848	66	35	0.53	-1.89	-0.92
A0A087W	Faim	23873	151	266	1.76	1.76	0.81
S4R2K3	Pcmtd1	319263	974	1,904	1.95	1.95	0.97
A0A0R4J	Znhit1	70103	19	41	2.22	2.22	1.15
P62509	Esrrg	26381	41	26	0.64	-1.56	-0.64
Q9CPP0	Npm3	18150	298	168	0.56	-1.77	-0.83
Q9EP89	Lactb	80907	1,075	707	0.66	-1.52	-0.60
P0DOV1	Mnda	381308	87	160	1.83	1.83	0.87
A2AS70	Mllt10	17354	8	14	1.60	1.60	0.68
E9QPI2	Nsun7	70918	151	88	0.58	-1.73	-0.79
P58069	Rasa2	114713	257	531	2.06	2.06	1.04
Q3UGY8	Arfgef3	215821	287	76	0.27	-3.76	-1.91

D3Z2H9	Tpm3-rs7	621054	4,191	6,443	1.54	1.54	0.62
P38533	Hsf2	15500	29	50	1.74	1.74	0.80
Q9JIM3	Ercc6l2	76251	140	76	0.55	-1.83	-0.87
P02468	Lamc1	226519	86	169	1.95	1.95	0.97
Q9JL35	Hmgn5	50887	4,452	2,365	0.53	-1.88	-0.91
P32043	Hoxc5	15424	123	52	0.42	-2.38	-1.25
G3X904	Zfp275	27081	58	96	1.67	1.67	0.74
Q9CQS2	Nop10	66181	621	1,021	1.65	1.65	0.72
Q8C9X6	Epc1	13831	10	15	1.53	1.53	0.61
J3QK52	Noc2l	57741	34	105	3.12	3.12	1.64
F7CT06	Unc5cl	76589	46	27	0.60	-1.68	-0.74
Q8BW49	Ttc12	235330	499	754	1.51	1.51	0.60
E9Q0B5	Fcgbp	215384	1,200	501	0.42	-2.40	-1.26
Q9ERU3	Znf22	67255	416	781	1.88	1.88	0.91
B2KF54	Ilrun	224647	119	66	0.55	-1.81	-0.86
Q80VM3	Ttc29	73301	28	51	1.82	1.82	0.87
P56382	Atp5f1e	67126	1,185	1,983	1.67	1.67	0.74
B1AV66	Yipf6	77929	18	27	1.55	1.55	0.63
A2AJT4	Pnisr	66625	540	901	1.67	1.67	0.74
Q9DB15	Mrpl12	56282	1,312	2,014	1.53	1.53	0.62
P56716	Rp1	19888	122	299	2.44	2.44	1.29
P14069	S100a6	20200	4,543	2,837	0.62	-1.60	-0.68
F6W322	Hnrnpm	76936	80	47	0.59	-1.70	-0.76
Q60972	Rbbp4	19646	1,934	1,224	0.63	-1.58	-0.66
Z4YN37	Kiz	228730	248	52	0.21	-4.77	-2.25
Q61781	Krt14	16664	1,918	1,152	0.60	-1.67	-0.74
P97364	Sephs2	20768	39	85	2.18	2.18	1.12
Q91V83	Tti1	75425	147	243	1.65	1.65	0.72
Q8R2Q8	Bst2	69550	616	1,054	1.71	1.71	0.77
Q9D6K7	Ttc33	67515	133	81	0.61	-1.65	-0.72
P41731	Cd63	12512	2,038	1,219	0.60	-1.67	-0.74
P23359	Bmp7	12162	57	30	0.53	-1.89	-0.92
O08807	Prdx4	53381	1,287	2,442	1.90	1.90	0.92
A6X8Z8	B4galt4	56375	48	75	1.55	1.55	0.64
A2AT02	Nsfl1c	386649	14	26	1.88	1.88	0.91
F6RVG2	Socs2	216233	27	46	1.72	1.72	0.78
Q8BG98	9130023H	100043133	12	21	1.71	1.71	0.77
Q8BIG7	Comtd1	69156	230	127	0.55	-1.81	-0.86
Q8BJG4	Mob3c	100465	14	22	1.57	1.57	0.65
Q99J08	Sec14l2	67815	350	131	0.38	-2.67	-1.41
Q8BJL0	Smarcal1	54380	161	102	0.63	-1.58	-0.66
F8WJ74	Gramd1a	52857	92	157	1.71	1.71	0.77
F6V8M6	Atxn2	20239	31	50	1.62	1.62	0.70
A0A2I3BF	Spcs1	69019	94	53	0.56	-1.78	-0.83
A3KGL9	Hmgn2	15331	558	1,203	2.16	2.16	1.11
A0A1Y7V	Tbc1d7	67046	199	69	0.35	-2.90	-1.54
Q9JKY7	Cyp2d22	56448	87	45	0.52	-1.92	-0.94
Q149B8	Perm1	74183	26	17	0.66	-1.52	-0.60

E9Q5M6	Cfap44	212517	151	232	1.54	1.54	0.62
A0A3B2W	Cyp39a1	56050	378	143	0.38	-2.64	-1.40
O08785	Clock	12753	297	181	0.61	-1.65	-0.72
Q3UJB3	Lrrc14b	432779	32	52	1.61	1.61	0.69
P63002	Tle5	14797	276	478	1.74	1.74	0.79
Q9CPW3	Mrpl54	66047	629	310	0.49	-2.03	-1.02
F6Q8D3	Atp11c	320940	233	138	0.59	-1.69	-0.76
Q922J3	Clip1	56430	179	74	0.41	-2.43	-1.28
Q8K5B2	Mcfd2	193813	17	65	3.79	3.79	1.92

p.value	is_sig
0.000	TRUE
0.000	TRUE
0.000	TRUE
0.001	TRUE
0.001	TRUE
0.001	TRUE
0.001	TRUE
0.001	TRUE
0.001	TRUE
0.001	TRUE
0.002	TRUE
0.002	TRUE
0.002	TRUE
0.003	TRUE
0.003	TRUE
0.004	TRUE
0.005	TRUE
0.005	TRUE
0.005	TRUE
0.006	TRUE
0.006	TRUE
0.007	TRUE
0.008	TRUE
0.008	TRUE
0.008	TRUE
0.008	TRUE
0.008	TRUE
0.009	TRUE
0.009	TRUE
0.009	TRUE
0.009	TRUE
0.010	TRUE
0.010	TRUE
0.011	TRUE
0.011	TRUE
0.011	TRUE
0.012	TRUE
0.013	TRUE
0.013	TRUE
0.013	TRUE
0.013	TRUE
0.014	TRUE
0.014	TRUE
0.015	TRUE
0.015	TRUE
0.015	TRUE

0.015	TRUE
0.015	TRUE
0.015	TRUE
0.015	TRUE
0.016	TRUE
0.016	TRUE
0.017	TRUE
0.018	TRUE
0.018	TRUE
0.019	TRUE
0.020	TRUE
0.020	TRUE
0.020	TRUE
0.020	TRUE
0.020	TRUE
0.021	TRUE
0.021	TRUE
0.021	TRUE
0.022	TRUE
0.022	TRUE
0.022	TRUE
0.022	TRUE
0.022	TRUE
0.023	TRUE
0.023	TRUE
0.023	TRUE
0.023	TRUE
0.024	TRUE
0.024	TRUE
0.024	TRUE
0.025	TRUE
0.025	TRUE
0.025	TRUE
0.026	TRUE
0.026	TRUE
0.026	TRUE
0.026	TRUE
0.026	TRUE
0.027	TRUE
0.027	TRUE
0.027	TRUE
0.027	TRUE
0.028	TRUE
0.028	TRUE
0.028	TRUE
0.029	TRUE
0.029	TRUE
0.030	TRUE

0.030	TRUE
0.030	TRUE
0.030	TRUE
0.031	TRUE
0.033	TRUE
0.034	TRUE
0.036	TRUE
0.036	TRUE
0.036	TRUE
0.036	TRUE
0.037	TRUE
0.038	TRUE
0.038	TRUE
0.038	TRUE
0.038	TRUE
0.038	TRUE
0.040	TRUE
0.040	TRUE
0.040	TRUE
0.040	TRUE
0.040	TRUE
0.040	TRUE
0.041	TRUE
0.042	TRUE
0.042	TRUE
0.042	TRUE
0.042	TRUE
0.042	TRUE
0.042	TRUE
0.042	TRUE
0.042	TRUE
0.043	TRUE
0.043	TRUE
0.043	TRUE
0.044	TRUE
0.044	TRUE
0.044	TRUE
0.044	TRUE
0.045	TRUE
0.045	TRUE
0.045	TRUE
0.046	TRUE
0.046	TRUE
0.046	TRUE
0.047	TRUE
0.047	TRUE
0.047	TRUE
0.047	TRUE

0.047	TRUE
0.047	TRUE
0.047	TRUE
0.048	TRUE
0.048	TRUE
0.048	TRUE
0.049	TRUE
0.049	TRUE
0.049	TRUE
